# Supplementary material for: Effect of Lean Red Meat from Beef (Pirenaica Breed) Versus Lean White Meat Consumption on Diet Quality: A Randomized-Controlled Crossover Study in Healthy Young Adults
Source: Nutrients. 2022 Dec 20;15(1):13. doi: 10.3390/nu15010013 (PMC9823384; doi:10.3390/nu15010013)
Supplement: Supplementary file 1 [file nutrients-15-00013-s001.zip › nutrients-2069810-supplementary.pdf]

PARTE 4 : CUESTIONARIO DE FRECUENCIA DE CONSUMO DE ALIMENTOS

Por favor, marcar una única opción para cada alimento.

Para cada alimento, marque el círculo que indica la frecuencia de consumo por término medio durante el año pasado. Se trata de tener en cuenta también la variación verano/invierno: por ejemplo, si tomas helados 4 veces/semana sólo durante los 3 meses de verano, el uso promedio al año es 1/semana.

43. I – LACTEOS *

Marca solo un óvalo por fila.

	NUNCA O CASI NUNCA	1-3 AL MES	1 A LA SEMANA	2-4 A LA SEMANA	5-6 A LA SEMANA	1 AL DÍA	2-3 AL DÍA	4-6 A DÍA
1.Leche entera (1 taza, 200 cc)								
2.Leche semidesnatada (1 taza, 200 cc)								
3.Leche desnatada (1 taza, 200 cc)								
4.Leche condensada (1 cucharada)								
5.Nata o crema de leche (1/2 taza)								
6.Batidos de leche (1 vaso, 200 cc)								
7.Yogurt entero (1, 125 gr)								
8.Yogurt desnatado (1, 125 gr)								
9.Petit suisse (1, 55 g)								
10.Requesón o cuajada (1/2 taza)								
11.Queso en porciones o cremoso (1, porción 25 g)								
12.Otros quesos: curados,								

semicurados (Manchego, Bola, Emmental…) (50 gr)								
13.Queso blanco o fresco (Burgos, cabra…) (50 gr)								
14.Natillas, flan, puding (1, 130 cc)								
15.Helados (1 cucurucho)								

44. II- HUEVOS, CARNES, PESCADOS(Un plato o ración de 100-150 gr,excepto cuando se indique otra cosa) *

Marca solo un óvalo por fila.

	NUNCA O CASI NUNCA	1-3 AL MES	1 A LA SEMANA	2-4 A LA SEMANA	5-6 A LA SEMANA	1 AL DÍA	2-3 AL DÍA	4-6 AL DÍA
16. Huevos de gallina (uno)	◯	◯	◯	◯	◯	◯	◯	◯
17. Pollo o pavo con piel (1 ración o pieza)	◯	◯	◯	◯	◯	◯	◯	◯
18. Pollo o pavo sin piel (1 ración o pieza)	◯	◯	◯	◯	◯	◯	◯	◯
19. Carne de ternera o vaca (1 ración)	◯	◯	◯	◯	◯	◯	◯	◯
20. Carne de cerdo (1 ración)	◯	◯	◯	◯	◯	◯	◯	◯
21. Carne de cordero (1 ración	◯	◯	◯	◯	◯	◯	◯	◯
22. Conejo o liebre (1 ración	◯	◯	◯	◯	◯	◯	◯	◯
23. Hígado (ternera, cerdo, pollo) (1 ración)	◯	◯	◯	◯	◯	◯	◯	◯
24. Otras vísceras (sesos, riñones, mollejas) (1 ración)	◯	◯	◯	◯	◯	◯	◯	◯
25. Jamón serrano (1	◯	◯	◯	◯	◯	◯	◯	◯

loncha, 30 g)								
26. Jamón York, jamón cocido (1 loncha, 30 g)								
27. Carnes procesadas (salchichón, chorizo, morcilla, mortadela, salchichas, butifarra, sobrasada, 50 g)								
28. Patés, foie-gras (25 g)								
29. Hamburguesa (una, 50 g), albóndigas (3 unidades)								
30. Tocino, bacon, panceta (50 g)								
31. Pescado blanco: mero, lenguado, besugo, merluza, pescadilla,… (1 plato, pieza o ración)								
32. Pescado azul: sardinas, atún, bonito, caballa, salmón, (1 plato, pieza o ración 130 g)								
33. Pescados salados:								

bacalao, mejillones,… (1 ración, 60 g en seco)								
34. Ostras, almejas, mejillones y similares (6 unidades)								
35. Calamares, pulpo, chipirones, jibia (sepia) (1 ración, 200 g)								
36. Crustáceos: gambas, langostinos, cigalas, etc. (4-5 piezas, 200 g)								
37. Pescados y mariscos enlatados al natural (sardinas, anchoas, bonito, atún) (1 lata pequeña o media lata normal, 50 g)								
38. Pescados y mariscos en aceite (sardinas, anchoas, bonito, atún) (1 lata pequeña o media lata normal, 50 g)								

45. III - VERDURAS Y HORTALIZAS(Un plato o ración de 200 g, excepto cuando se indique) *

Marca solo un óvalo por fila.

	NUNCA O CASI NUNCA	1-3 AL MES	1 A LA SEMANA	2-4 A LA SEMANA	5-6 A LA SEMANA	1 AL DÍA	2-3 AL DÍA	4-6 AL DÍA
39. Acelgas, espinacas	⬭	⬭	⬭	⬭	⬭	⬭	⬭	⬭
40. Col, coliflor, brócoles	⬭	⬭	⬭	⬭	⬭	⬭	⬭	⬭
41. Lechuga, endivias, escarola (100 g)	⬭	⬭	⬭	⬭	⬭	⬭	⬭	⬭
42. Tomate crudo (1, 150 g)	⬭	⬭	⬭	⬭	⬭	⬭	⬭	⬭
43. Zanahoria, calabaza (100 g)	⬭	⬭	⬭	⬭	⬭	⬭	⬭	⬭
44. Judías verdes	⬭	⬭	⬭	⬭	⬭	⬭	⬭	⬭
45. Berenjenas, calabacines, pepinos	⬭	⬭	⬭	⬭	⬭	⬭	⬭	⬭
46. Pimientos (150 g)	⬭	⬭	⬭	⬭	⬭	⬭	⬭	⬭
47. Espárragos	⬭	⬭	⬭	⬭	⬭	⬭	⬭	⬭
48. Gazpacho andaluz (1 vaso, 200 g)	⬭	⬭	⬭	⬭	⬭	⬭	⬭	⬭
49. Otras verduras (alcachofa,	⬭	⬭	⬭	⬭	⬭	⬭	⬭	⬭

puerro, cardo, apio)								
50. Cebolla (media unidad, 50 g)								
51. Ajo (1 diente)								
52. Perejil, tomillo, laurel, orégano, etc. (una pizca)								
53. Patatas fritas comerciales (1 bolsa, 50 g)								
54. Patatas fritas caseras (1 ración, 150 g)								
55. Patatas asadas o cocidas								
56. Setas, níscalos, champiñones								

46. IV – FRUTAS Y FRUTOS SECOS(una pieza o ración) *

Marca solo un óvalo por fila.

	NUNCA O CASI NUNCA	1-3 AL MES	1 A LA SEMANA	2-4 A LA SEMANA	5-6 A LA SEMANA	1 AL DÍA	2-3 AL DÍA	4-6 [illegible] DÍ[illegible]
57. Naranja (una), pomelo (uno), mandarinas (dos)								
58. Plátano (uno)								
59. Manzana o pera (una)								
60. Fresas/fresones (6 unidades, 1 plato postre)								
61. Cerezas, picotas, ciruelas (1 plato de postre)								
62. Melocotón, albaricoque, nectarina (una)								
63. Sandía (1 tajada, 200-250 g)								
64. Melón (1 tajada, 200-250 g)								
65. Kiwi (1 unidad, 100 g)								
66. Uvas (un racimo, 1 plato postre)								
67. Aceitunas (10 unidades)								
68. Frutas en								

almíbar o en su jugo (2 unidades)								
69. Dátiles, higos secos, uvas-pasas, ciruelas-pasas (150 g)								
70. Almendras, cacahuetes, avellanas, pistachos, piñones (30 g)								
71. Nueces (30 g)								

47. ¿Cuántos días a la semana tomas fruta como postre? *

Marca solo un óvalo.

- 1
- 2
- 3
- 4
- 5
- 6
- 7

48. V-LEGUMBRES y CEREALESUn plato o ración (150 g) *

Marca solo un óvalo por fila.

	NUNCA O CASI NUNCA	1-3 AL MES	1 A LA SEMANA	2-4 A LA SEMANA	5-6 A LA SEMANA	1 AL DÍA	2-3 AL DÍA	4-6 AL DÍA
73. Lentejas (1 plato, 150 g cocidas)	⬭	⬭	⬭	⬭	⬭	⬭	⬭	⬭
74. Alubias (pintas, blancas o negras) (1 plato, 150 g	⬭	⬭	⬭	⬭	⬭	⬭	⬭	⬭
75. Garbanzos (1 plato, 150 g cocidos)	⬭	⬭	⬭	⬭	⬭	⬭	⬭	⬭
76. Guisantes, habas (1 plato, 150 g cocidos)	⬭	⬭	⬭	⬭	⬭	⬭	⬭	⬭
77. Pan blanco, pan de molde (3 rodajas, 75 g)	⬭	⬭	⬭	⬭	⬭	⬭	⬭	⬭
78. Pan negro o integral (3 rodajas, 75 g)	⬭	⬭	⬭	⬭	⬭	⬭	⬭	⬭
79. Cereales desayuno (30 g)	⬭	⬭	⬭	⬭	⬭	⬭	⬭	⬭
80. Cereales integrales: muesli, copos avena, all-bran (30 g)	⬭	⬭	⬭	⬭	⬭	⬭	⬭	⬭
81. Arroz	⬭	⬭	⬭	⬭	⬭	⬭	⬭	⬭

blanco (60 g en crudo)								
82. Pasta: fideos, macarrones, espaguetis, otras (60 g en crudo)								
83. Pizza (1 ración, 200 g)								

49. VI- ACEITES Y GRASASUna cucharada sopera o porción individual.Para freír, untar, mojar en el pan, para aliñar, o paraensaladas, utilizas en total: *

Marca solo un óvalo por fila.

	NUNCA O CASI NUNCA	1-3 AL MES	1 A LA SEMANA	2-4 A LA SEMANA	5-6 A LA SEMANA	1 AL DÍA	2-3 AL DÍA	4-6 AL DÍA
84. Aceite de oliva (una cucharada sopera)								
85. Aceite de oliva extra virgen (una cucharada sopera)								
86. Aceite de oliva de orujo (una cucharada sopera)								
87. Aceite de maíz (una cucharada sopera)								
88. Aceite de girasol (una cucharada sopera)								
89. Aceite de soja (una cucharada sopera)								
90. Mezcla de los anteriores (una cucharada sopera)								

91. Margarina (porción individual, 12 g)								
92. Mantequilla (porción individual, 12 g)								
93. Manteca de cerdo (10 g)								

50. VII - BOLLERÍA Y PASTELERÍA *

Marca solo un óvalo por fila.

	NUNCA O CASI NUNCA	1-3 MES	1 A LA SEMANA	2-4 A LA SEMANA	5-6 A LA SEMANA	1 AL DÍA	2-3 AL DÍA	4-6 AL DÍA
94. Galletas tipo maría (4-6 unidades, 50 g)								
95. Galletas integrales o de fibra (4-6 unidades, 50 g)								
96. Galletas con chocolate (4 unidades, 50 g)								
97. Repostería y bizcochos hechos en casa (50 g)								
98. Croissant, ensaimada, pastas de té u otra bollería industrial comercial ... (uno,50g)								
99. Donuts (uno)								
100. Magdalenas (1-2 unidades)								
101. Pasteles (uno, 50 g)								

102. Churros, porras y similares (1 ración, 100 g)								
103. Chocolates y bombones (30 g)								
104. Cacao en polvo-cacaos solubles (1 cucharada de postre)								
105. Turrón (1/8 barra, 40 g)								
106. Mantecados, mazapán (90 g)								

51. VIII – OTROS ALIMENTOS *

Marca solo un óvalo por fila.

	NUNCA O CASI NUNCA	1-3 AL MES	1 A LA SEMANA	2-4 A LA SEMANA	5-6 A LA SEMANA	1 AL DÍA	2-3 AL DÍA	4-6 AI DÍA
107. Croquetas, buñuelos, empanadillas, precocinados (una)								
108. Sopas y cremas de sobre (1 plato)								
109. Mostaza (una cucharadita de postre)								
110. Mayonesa comercial (1 cucharada sopera = 20 g)								
111. Salsa de tomate frito, ketchup (1cucharadita)								
112. Picante: tabasco, pimienta, pimentón (una pizca)								
113. Sal (una pizca)								
114. Mermeladas (1 cucharadita)								
115. Azúcar (1 cucharadita)								
116. Miel (1								

cucharadita)								
117. Snacks distintos de patatas fritas: gusanitos, palomitas, maíz, etc. (1 bolsa, 50 g)								

52. IX – BEBIDAS *

Marca solo un óvalo por fila.

	NUNCA O CASI NUNCA	1-3 AL MES	1 A LA SEMANA	2-4 A LA SEMANA	5-6 A LA SEMANA	1 AL DÍA	2-3 AL DÍA	4-6 AL DÍA
119. Bebidas carbonatadas con azúcar: bebidas de cola, limonadas, tónicas, etc. (1 botellín, 200 cc)								
120. Bebidas carbonatadas bajas en calorías, bebidas light (1 botellín, 200 cc)								
121. Zumo de naranja natural (1 vaso, 200 cc)								
122. Zumos naturales de otras frutas (1 vaso, 200 cc)								
123. Zumos de frutas en botella o enlatados (200 cc)								
124. Café descafeinado (1 taza, 50 cc)								
125. Café (1 taza, 50 cc)								
126. Té (1 taza, 50 cc)								

127. Mosto (100 cc)								
128. Vaso de vino rosado (100 cc)								
129. Vaso de vino moscatel (50 cc)								
130. Vaso de vino tinto joven, del año (100 cc)								
131. Vaso de vino tinto añejo (100 cc)								
132. Vaso de vino blanco (100 cc)								
133. Vaso de cava (100 cc)								
134. Cerveza (1 jarra, 330 cc)								
135. Licores, anís o anisetes ... (1 copa, 50 cc)								
136. Destilados: whisky, vodka, ginebra, coñac (1 copa, 50 cc)								

53. ¿A qué edad empezaste a beber alcohol (vino, cerveza o licores), incluyendo el que tomas con las comidas con regularidad (más de siete "bebidas" a la semana)? *

54. ¿Cuántos años has bebido alcohol con regularidad (más de siete bebidas a la semana)? *

55. MARCAS DE LOS SUPLEMENTOS DE VITAMINAS O MINERALES O DE LOS PRODUCTOS DIETÉTICOS

56. Frecuencia de consumo *

Marca solo un óvalo por fila.

	NUNCA O CASI NUNCA	1-3 MES	1 A LA SEMANA	2-4 A LA SEMANA	5-6 A LA SEMANA	1 AL DÍA	2-3 AL DÍA	4-6 AL DÍA
1er suplemento	⬭	⬭	⬭	⬭	⬭	⬭	⬭	⬭
2º suplemento	⬭	⬭	⬭	⬭	⬭	⬭	⬭	⬭

Este contenido no ha sido creado ni aprobado por Google.

Google Formularios
